# Evaluating Heat Stress Effects on Growth in Tunisian Local Kids: Enhancing Breeding Strategies for Arid Environments

**DOI:** 10.3390/ani14192846

**Published:** 2024-10-03

**Authors:** Ahlem Atoui, Sghaier Najari, Clara Diaz, Manuel Ramón, Mohamed Ragab, Aicha Laaroussi, Mouldi Abdennebi, Maria Jesus Carabaño

**Affiliations:** 1 Laboratory of Livestock and Wildlife, Institute of Arid Regions (IRA), Medenine 4119, Tunisia; najarisghaier@yahoo.fr (S.N.); aicha.laarousi.25@gmail.com (A.L.); abdennebim@yahoo.fr (M.A.); 2 Depto de Mejora Genética Animal, INIA-CSIC, Ctra de La Coruña Km 7.5, 28040 Madrid, Spain; 3 Higher Institute of Human Sciences of Medenine (ISSHM), University of Gabes, Gabes 6029, Tunisia; cdiaz@inia.csic.es (C.D.); manuel.ramon@inia.csic.es (M.R.); moha.ragab@hotmail.com (M.R.); mjc@inia.csic.es (M.J.C.); 4 Poultry Production Department, Faculty of Agriculture, Kafrelsheikh University, Kafrelsheikh 33516, Egypt

**Keywords:** heat stress, kid growth, individual heat tolerance, weight loss

## Abstract

**Simple Summary:**

In the arid south of Tunisia, goats are favored by herders due to their demonstrated resilience. In the present study, the effect of heat stress on the growth of Tunisian local kids from birth to weaning age was evaluated, with body weight being considered as an important economic trait in goat production. A significant negative impact of heat stress on goat performance, evidenced by reductions in body weight under the conditions of moderate and acute heat stress, was obtained. Such results are crucial for informing selective breeding strategies aimed at enhancing livestock resilience to environmental stressors like heat, particularly in arid regions where such conditions are prevalent.

**Abstract:**

This study evaluates the impact of thermal load on the weights of Tunisian local kids using 24 models with cubic and quadratic Legendre polynomials, based on daily temperatures (Tmin, Tmax, and Tavg) on the day of weight recording and averaged over 7, 14, and 21 days before weighing. The deviance information criterion (DIC) consistently shows that cubic polynomial models offer a better fit than quadratic models, highlighting their superior accuracy in studying the effects of thermal load on kid weights. The models with the best fit utilized average or maximum temperatures over 14 or 21 days. The patterns of response were similar across the temperature variables and periods, showing a stable weight response at lower temperatures (thermoneutral region) followed by a decline as the temperatures increased. The weight loss was −125 g/°C beyond the moderate heat stress threshold (Tavg21 = 17.7 °C) and −450 g/°C beyond the severe heat stress threshold (Tavg21 = 25.3 °C) for Tavg21. The heat stress thresholds for moderate heat stress (HS1) were 8.6 °C for Tmin14, 27.4 °C for Tmax14, and 18.6 °C for Tavg14; moreover, for acute heat stress (HS2), they were 17.2 °C for Tmin14, 32.4 °C for Tmax14, and 25.5 °C for Tavg14. High variability in individual responses was observed, with differences in the slope of response ranging from 2.0 kg/°C for moderate heat stress to around 3.0 kg/°C for severe heat stress for Tavg. The correlations between the weights under different temperatures were low, indicating that rankings based on weight could change with varying heat conditions. The animals with larger weight levels generally demonstrated better heat tolerance, and those with good heat tolerance under moderate conditions were also likely to have good tolerance under severe conditions.

## 1. Introduction

In the arid south of Tunisia, as well as in other arid areas of the world, the climate is harsh and dry. Rainfall is scarce, averaging only 200 mm annually. Summers are exceptionally hot and parched, with temperatures soaring up to 47 °C [1], posing considerable challenges for agriculture and water management. In these harsh environments, goats are favored by herders due to their demonstrated resilience in coping with multifaceted stressors such as heat, water scarcity, and limited forage availability, surpassing that of sheep and cattle in adaptation [2]. Najari [3] highlights the specific traits of goats that aid them in coping with environmental challenges across ecosystems. These include their small body size, enabling efficient escape from high radiant heat, along with lower absolute requirements for energy, water, and home range and their capacity to digest dry matter and efficiently recycle nitrogen [3]. Despite of their good adaptation to harsh environmental conditions, because of the small body size and low productivity of the local goats, farmers may opt for foreign selected breeds, which may not possess the same level of adaptation to challenging conditions, posing a threat to the preservation of local genetic resources. Alternatively, local populations could be selected to improve productivity, which might also compromise the adaptation to harsh conditions. Previous studies [2,4] have dealt with the search for optimal breeding tools to improve kids’ growth in this breed. However, no knowledge exists regarding variability in adaptation traits and the relationship with the selection objective aiming to improve productivity, mainly through growing potential. This study aims to characterize the overall and individual response to high temperatures in the weight of kids before weaning in the Tunisian goat population. In addition, the relationship between growing potential and heat tolerance will be estimated. We considered heat tolerance as an indicator of adaptation because of the easy access to daily temperatures from a close-by weather station and because the reaction of growth to changes in temperature is expected to reflect not only the impact of ambient temperature itself on the animal, but also of the impact that high temperatures have on feed availability and feed quality. The results of this study should provide helpful information to define the overall breeding goal in this breed, which is to improve productivity without losing adaptation to the environment.

## 2. Material and Methods

### 2.1. Data and Animal Management

The animals studied are part of the experimental goat herd located at the Arid Areas Institute of Medenine, Tunisia (33°30′ N and 10°40′ E). Situated in southeastern Tunisia, between the Matmata Mountains and the Mediterranean Sea, this region experiences an arid continental Mediterranean climate. It is characterized by irregular precipitation, averaging around 200 mm annually. Summer, known for its scorching temperatures, is the hottest and driest season, with maximum temperatures reaching up to 47 °C [2]. The animals were managed within an extensive production system, with natural pasture serving as the primary food source. The quantity and quality of the pasture fluctuated significantly throughout the year and from year to year. With the dry season, the quantity and quality of the pasture decreased, and supplemental feeding had to be provided. The primary mating period of the local goats occurred between June and August. If a doe remained non-pregnant during the initial mating period, it was then moved to the group designated for mating during the subsequent period (October–November, corresponding to births in spring). The kidding season started in October and extended until February, with a concentration of births in November and December. On average, the suckling period lasted for 120 days. Female kids were initially mated between 12 and 18 months, depending on their season of birth and their body condition.

The weight records used in this study were collected from 1998 to 2019. Throughout the analysis period, the kids were weighed at intervals of 3 weeks from birth until weaning.

The daily maximum (Tmax), minimum (Tmin), and average (Tavg) temperatures were provided by the state meteorological agency for the period of weight recording. The file containing the weight recording information was merged with the temperature data file by the date of recording. The temperatures on the day of recording, plus the average values of the daily values for the 7, 14, and 21 days prior to the date of recording were obtained for each recorded weight.

After editing the weight and temperature files to eliminate records with missing critical information or with abnormal values and merging them with the meteorological data, 6886 weights recorded in 971 kids were available for this study.

### 2.2. Statistical Analyses

#### 2.2.1. Models

Mixed models were used to estimate the average or population response in weight to changes in heat load and the individual response deviations. Heat load was measured by the temperature on the day of weighing or the average of temperatures during 7/14/21 days before weighing.

The general model equation was as follows:

(1) $$y_{\mathrm{ikj}}={\mathrm{SEF}}_{i}+\sum_{j=0}^{q=2,3}b_{j}x_{j}+\sum_{j=0}^{2}\alpha_{kj}x_{j}+e_{ikl}$$

where SEF are the systematic environmental effects affecting the weight of the kids but not related with the effect of heat load, measured by temperature. In this case, SEF contained the effect of the year of birth of the kid, the age (11 classes, one per month of age) and weight of the dam at kidding (3 classes, 1 = 14.5 to 24.9 kg; 2 = 26 to 32; 3 = 32.5–45 kg), the interaction sex of the kid (male or female), the type of birth (single, double, or more) as class effects, and the age of the kid at weighing using a quadratic polynomial nested to the corresponding sex-type of birth class. The x_j_ are covariables, including the heat load linked to each weight, and the bj and $\alpha_{kj}$ are regression coefficients to describe the overall and individual response of weight to changes in heat load. Quadratic and cubic polynomials were used alternatively to fit the overall curve of response, while only quadratic polynomials were used to fit the individual curve deviations.

The (co)variance structure was as follows:

$$var=\left(\times \right)I,\;var\left(e\right)=D$$

where

α is a vector containing the three random regression coefficients for the intercept ($\alpha_{0j}$), linear ($\alpha_{1j})$, and quadratic $\;(\alpha_{2j}$) coefficients for each kid j;

G_o_ is a 3 × 3 matrix of (co)variances between random regression coefficients;

e is the vector of residual effects;

D is a diagonal matrix with heterogeneous variances corresponding to 5 age classes (class 1, from 1 to 15 days of age; classes 2 to 4 comprising 30 subsequent days; and class 5 from day 107 to day 150 of age at recording), to account for the expected increase in variance of weight as age increases, associated to scale effects.

Heat load was measured through the daily values of temperature, considering the minimum (Tmin), maximum (Tmax), and average (Tavg) temperature on the day of weighing (0 = recording day), as well as the average of those values observed in another three periods prior to weight recording (7, 14, 21 days). In total, 24 models were solved to compare the effect of the polynomial degree (quadratic vs. cubic) used to define the overall response curve and the 12 combinations of the three daily temperatures and the four periods of time. Package MCMCglmm of R, which relies on Bayesian estimation, was used to solve the models. For all models, 10,000 samples (including 2500 considered as burn-in samples) were obtained for each parameter to obtain the posterior modes that were used as estimates for the model unknowns. Convergence was checked via visual inspection.

In addition to the 24 models, a model that did not include the effect of heat load on weights was fitted in order to provide a way to determine the relevance of the heat load effect. The goodness of fit of the alternative models was compared using the deviance information criterion (DIC) provided by the MCMCglmm package.

#### 2.2.2. Curves of Response to Increasing Heat Loads in Weights

The population or average response in weight of kids from the Tunisian goat population to an increase in temperature was obtained using the following expression:

wgt(T) = **Zb**

where wgt(T) represents values of the expected average age-adjusted weight as a function of temperature (T), **b** is the vector of the regression coefficients b_j_ in [1], and **Z** is a matrix containing the Legendre polynomial covariables for each T within the range of observed temperatures.

The target parameters used to characterize the response were HS thresholds and losses in weight associated with HS. The threshold temperatures for the appearance of HS were obtained from change points in the polynomial fit, and losses due to HS were obtained from the slopes of the curve between change points. The segmented package of R (Muggeo, 2008) was used for this purpose. The method proposed by Muggeo [5] provides a linear segmented fit from a vector of x/y values for a pre-stablished number of change points. We used two in this study in order to consider a moderate (HS1) and a more acute (HS2) heat stress.

Individual deviations from the average response curve for each kid were obtained from the following:

$${\mathrm{wgt}}_{j}(T)=Z\alpha_{j}$$

where wgt_j_(T) is the predicted individual deviation of age-adjusted weight at successive values of temperature for kid j, and $\alpha_{j}$ contains the $\alpha_{kj}$ regression coefficients in [1] for individual j.

The target parameters for individual kids were the intercept coefficient, interpreted as the basal level of weight, not influenced by the change in temperature, and the slopes of change in weight within the HS region (determined previously by the change points in the population response). Given the underlying assumption of a zero mean for random effects in mixed models, the estimated slopes for individual kids deviate around zero. A positive slope of change per degree of increase in temperature will result in lower losses in weight for the individual, with respect to the average population response, and in the animal being considered as heat tolerant. On the other hand, animals showing large, negative individual slopes will show a steeper rate of loss in weight with respect to the average, and the animal will be considered as heat susceptible.

Estimates of individual slopes were obtained through the calculation of the first derivative of the response curve for each individual as follows:

$${\mathrm{Slp}}_{j}(T)=C\prime \alpha_{j}$$

where Slp_j_(T) represents the slope of the response curve for individual j at a temperature = T, and C′ is the matrix of first derivatives of the regression co-variables with respect to temperature, evaluated at HS1 and HS2.

#### 2.2.3. (Co)Variability for Individual Weight and Slope of Weight Loss under Heat Stress

From the estimated (co)variance components among random regression coefficients, G_o_, changes in weight variance along the temperature range and correlations between individual weight records obtained under different temperatures were obtained as follows:

$$\mathrm{Var}(Z\alpha )={\mathrm{ZG}}_{o}Z\prime$$

These estimates provide information about gain or loss in the variability of the trait under HS and also about expected changes in the ranking of animals for weight along the temperature scale, which would be associated to differences in heat tolerance.

Similarly, the (co)variability for individual slopes of response to HS was obtained from the following expression:

$$\mathrm{Var}\;(C\prime \alpha )={C\prime G}_{o}C$$

In addition, the covariance between intercepts ($\alpha_{0}$) and slopes of loss (Slp(T)) was obtained in order to define the relationship between the individual growth (measured by the intercept) and heat tolerance (measured by the slope of weight loss under heat stress).

## 3. Results

Summary statistics depicting the distributions of weight, age, and climate data characterizing the environmental conditions during the period of this study are presented in Table 1.

The weights ranged from 1.4 kg on the day after birth to 23.4 kg at weaning. Ninety percent of the kids had three weights or more recorded. The temperatures on the day of weighing ranged from 0 °C to 36 °C. In this respect, we have to mention that the latest weaning date was in June, resulting from the breeding management decision to avoid lactating periods during the hardest days of the summer. It is also worth noting that the distribution for the same temperature (minimum, average, or maximum) was different for the four periods considered to calculate the heat load variable (0, 7, 14, and 21). As expected, the means for the Tmin values over the periods became lower as the period length increased, while the means for Tmax decreased. The standard deviations decreased as the length of the periods increased. Taking into account the difference in scales of the different heat loads will be relevant when comparing the results from alternative models.

### 3.1. Statistical Model Comparison

**Figure 1:**
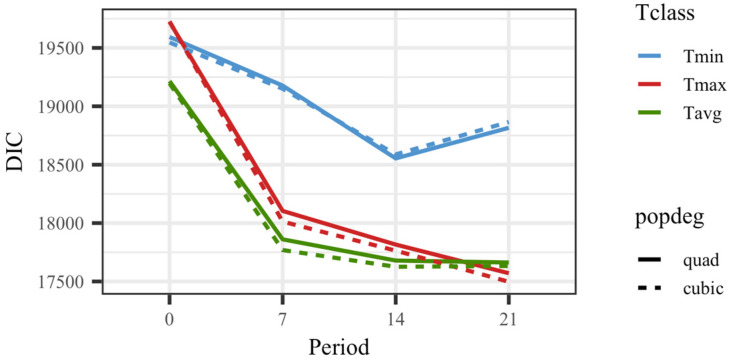
Deviance information criteria (DIC) for models fitting quadratic (quad) or cubic Legendre polynomials on average of minimum (Tmin), maximum (Tmax), and average (Tavg) daily temperatures on the day of weighing (0), 7, 14, and 21 days prior to the date of weighing.

All models including temperature showed a superior goodness of fit to the model that did not consider temperature as an explanatory variable for weights. The DIC value was 21,396.4 for the model without temperature vs. 19,593.3 for the model considering Tmin0 (the worst fitting model for those including temperature). Figure 1 shows DIC values for all 24 models that included the thermal load as an effect on kid weights. The models fitting a cubic polynomial for the overall response showed slightly better goodness of fit than the models fitting a quadratic response. The average daily values for temperature rendered better results than maximum or minimum values in general. Overall, the models showing the best goodness of fit were those based on the average or maximum temperature in a period of 14 or 21 days before the day of weighing. Since the models fitting temperatures on the date of recording showed substantially worse fitting ability, no results from those models will be further discussed.

### 3.2. Overall Population Response

**Figure 2:**
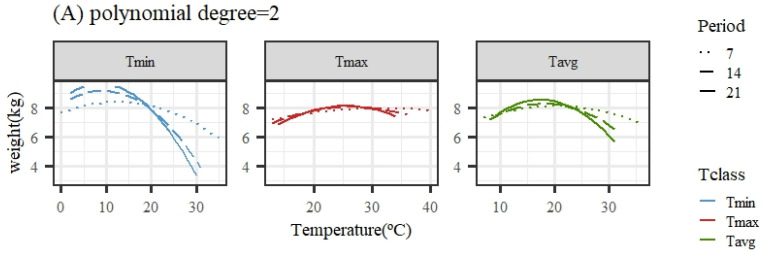

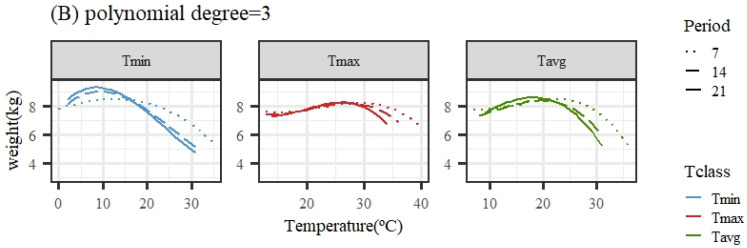
Expected change in weight for different values of the average of minimum (Tmin), maximum (Tmax), and average (Tavg) daily temperatures during 7, 14, and 21 days prior to the date of weighing when using quadratic (**A**) or cubic (**B**) polynomial functions.

Figure 2 illustrates the expected loss in weight associated with HS obtained from the average population response using quadratic or cubic polynomial functions. The patterns of response for all temperature variables and periods were similar, showing a close-to-flat response for the lower temperatures of the range (interpreted as the thermoneutral region) followed by a decline in the expected weight (adjusted for age in the models of analyses) as the temperature increased. An exception was the pattern for Tmax under the quadratic polynomial function, with nearly no decline along the whole range of temperatures.

Given that the quadratic functions showed a worse goodness of fit ability, the estimates of change points and slopes of change along the weight response to temperature are only shown for cubic polynomials in Table 2. For the same temperature variable (Tmin, Tmax, or Tavg), the heat stress thresholds lowered as the number of days in the considered period to measure the temperatures prior to weight recorded increased, due to the different scales of temperatures averaged over periods differing in length (as mentioned previously), making a direct comparison across periods somehow cumbersome. Taking the results from the 14-day period, the period showing better fitting ability for all temperature variables, the moderate heat stress (HS1) threshold estimate occurred when the average daily temperature was 8.6/27.4/18.6 °C for Tmin14/Tmax14/Tavg14. Acute heat stress (HS2) occurred when the average daily temperature during the period was 17.2/32.4/25.5 °C for Tmin14/Tmax14/Tavg14.

The results in Table 2 for the slopes of change after the HS thresholds show that HS was detected either when the animals were exposed to high temperatures during a short time (7 days) before the day of weighing the animals or to the cumulative effect of exposing the animals to heat during 2 or 3 weeks before the weight was recorded.

The largest estimated loss in weight was obtained for Tavg21, with a slope of loss of −125 g/°C beyond the moderate HS threshold (Tavg21 = 17.7 °C) and −450 g/°C beyond the more acute HS threshold (Tavg21 = 25.3 °C), emphasizing the significant impact of sustained high average temperatures. On the other hand, the variable capturing the smallest losses in weight was Tmin7, with slopes values of −75 g/°C for moderate (Tmin7 = 13.6 °C) and −228 g/°C for severe (Tmin7 = 25.4 °C) HS.

### 3.3. Individual Response

**Figure 3:**
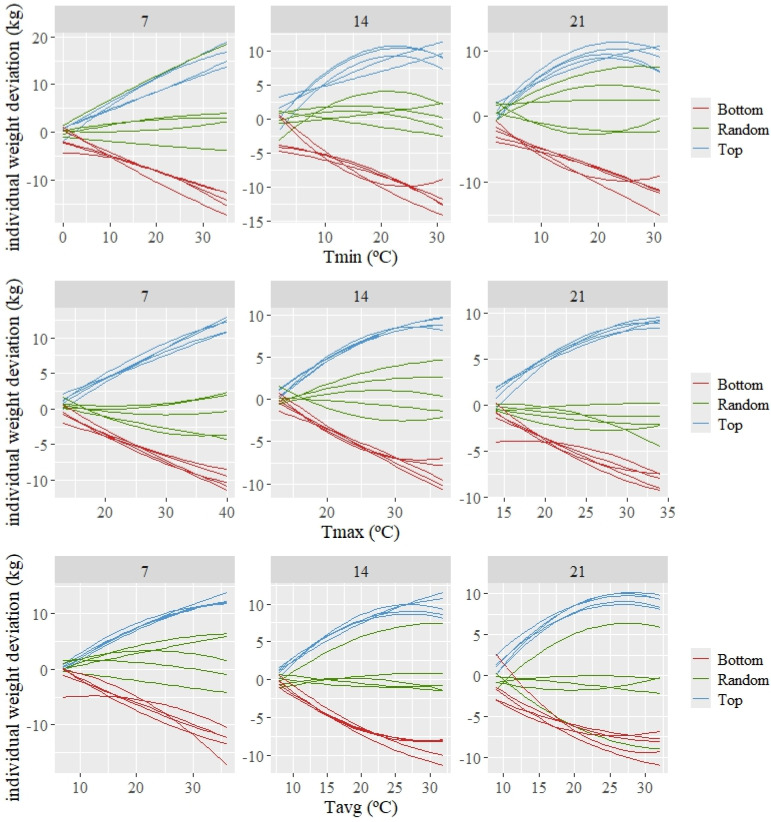
Predicted individual weight deviations to changes in the average of minimum (Tmin), maximum (Tmax), and average (Tavg) during 7, 14, and 21 days prior to weighing for the 5 best (Top), worst (Bottom), and random kids, according to the estimated values for the intercept.

The estimated individual patterns of response (which are deviations from the average or population response) are shown in Figure 3 for three groups of five animals composed of the best, random, and worst kids according to the estimated value for the intercept (basal level of weight). A variety in patterns of response can be observed among the 15 animals shown in this figure. The top individuals, which show a tendency to have higher weights than the average as the temperature increases, demonstrate a high tolerance to increasing average temperatures. The opposite can be observed for the bottom individuals (those with lower basal weight), for which their weight decreases more than the average as the temperature increases.

Table 3 shows the distribution of the estimated individual slopes at two temperatures representing moderate and severe HS for all of the individuals participating in the analyses. The temperature values associated with these two levels of HS differed for the different temperature variables and periods according to the HS thresholds shown in Table 2.

High variability in estimated individual response to HS, namely in heat tolerance, was observed for all temperature measures and periods. For example, for the variable average temperature in a 14-day period prior to weighing, the differences in the slope of response between the most and least tolerant animals was 2.0 kg/°C for the moderate HS1 and around 3.0 kg/°C under acute HS2, while, for the same variable and period, the differences between the 10 and 90 percentiles ranged between 0.8 kg/°C and 1 kg/°C for moderate and acute HS, respectively.

### 3.4. (Co)Variability for Individual Weight and Slope of Weight Loss under Heat Stress

**Figure 4:**
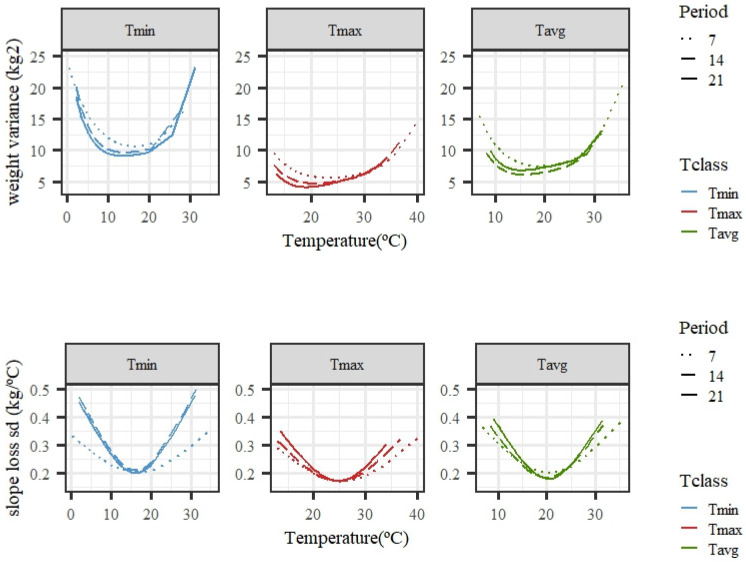
Estimated variance for weights (top row) and standard deviation of slopes of change in weight (bottom row) along the temperature scale under models fitting individual cubic Legendre polynomials on the average of minimum (Tmin), maximum (Tmax), and average (Tavg) daily temperatures during 7, 14, and 21 days prior to the date of weighing.

Figure 4 shows the estimated changes in weight variance and slope standard deviation along the temperature scale for models fitting individual cubic Legendre polynomials. The variability of weights was larger under high temperatures, especially for Tmax and Tavg, while, for Tmin, higher variability was also observed for the lowest values, suggesting a variation in tolerance to low temperatures. The variability of the slope of the curves under low or high temperatures was larger than that observed for the intermediate temperatures. Close-to-null variability within the thermoneutral region was expected, because the response in weight to an increase in temperature should be flat, i.e., a close-to-zero slope. The trends were consistent regardless of the length of the period considered (7, 14, or 21 days), although there were slight differences in the exact shape and steepness of the curves. Tmin showed a rather higher variability of slopes than Tmax or Tavg for the average of temperatures in 14 or 21 days. 

**Figure 5:**
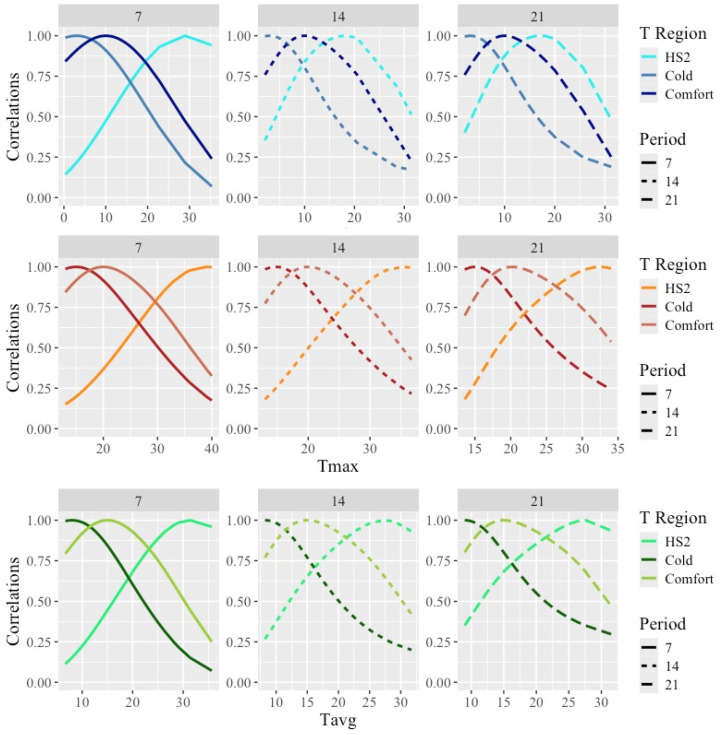
Estimated correlations between weights obtained under temperatures representing acute heat stress (HS2), cold, and comfort and weights along the temperature scale under models fitting individual cubic Legendre polynomials on the average of minimum (Tmin), maximum (Tmax), and average (Tavg) daily temperatures during 7, 14, and 21 days prior to the date of weighing. Temperatures representing HS2 can be found in Table 4 for combinations of temperature type and period. Temperatures representing Cold/Comfort were 3/10 °C for Tmin, 15/21 °C for Tmax, and 8/16 °C for Tavg.

The estimated correlations between the weights obtained under acute heat stress, cold, or comfortable temperatures and the weights recorded along the whole range of temperatures are shown in Figure 5.

As expected from the use of smooth covariance functions, correlations between the weights were close to one for the weights taken under similar temperatures and decreased as temperatures differed from the HS1 and HS2 temperatures. Overall, the estimated correlations between the weights taken under HS and the weights under comfort were quite low, with lower values for Tmax (with minimum values for correlations with weights under the lower temperatures of 0.2) and larger values for Tmin (with minimum ranging from 0.2 for Tmin7 to 0.8 for Tmin21). Thus, large differences in the ranking of animals by weight under hot vs. comfortable or cold weather would be expected.

The relationship between the weight level (intercepts) and heat tolerance (slopes) is described in Table 4, where correlations between the predicted values for the intercepts and the slopes of change in weight under moderate or severe HS are shown. A positive correlation between the intercept and the slope means that the best animals for weight potential will tend to also be the best for heat tolerance. For all temperature variables and periods, rank correlations were high for moderate HS, ranging from 0.92 for Tmin7 to 0.74 for Tmax7 and Tmax14. Those correlations were more variable for severe HS, ranging from 0.8 for Tmin21 to 0.12 for Tavg21. Overall, the animals that showed a higher weight level tended to be more heat tolerant than the average, and vice versa. The correlations between the estimated slopes under moderate and severe HS are also shown in Table 4. A positive rank correlation between the slopes for moderate or severe heat stress means that the severity of heat stress does not modify the ranking of the animals, according to the heat tolerance indicator. These correlations were over 0.6 for all cases, except for the correlation between the two heat tolerance levels for Tavg21, which showed a value of 0.31. In general, the animals that show good levels of heat tolerance under moderate HS are expected to show good levels of heat tolerance under more severe HS.

## 4. Discussion

In the present study, the effect of heat stress on the growth of Tunisian local kids from birth to weaning age was evaluated, with body weight being considered as an important economic trait in goat production. Moreover, we investigated the HS effects using different definitions of heat load (with respect to lag effects and the use of daily maximum, average, or minimum values) to propose the most appropriated class to be used. The average of the average daily temperatures provided the best fit of the available data and captured the largest losses in weight associated with the effect of increasing temperature. The daily maximum temperatures provided similar results to the daily averages in terms of goodness of fit but yielded smaller slopes of decay than the daily average temperatures. The maximum temperatures were much less variable (CV ranged from 23% to 27%) than the minimum (CV ranged from 49% to 61%) or average temperatures (CV ranged from 30% to 33%) and, although they correlated with the minimum or average temperatures, they did not fully reflect the thermal environment. The minimum temperatures were less relevant in terms of describing the effects of HS on weights (they showed the worst goodness of fit and smallest slopes of decay in weight); however, they still reflected negative effects of high minimum temperatures during the night. Overall, the average temperatures, accounting for both the maximum and minimum values, might be expected to be a better measure of the impact of environmental heat load on animals. The average temperature over a two-to-three-week period provided the best fit, indicating the need to account for accumulated heat loads in order to determine the effect of HS on growth.

The results obtained in this study can be used as a first approach to quantify the expected loss in weight or growth associated to heat stress under the temperatures that kids experienced during the pre-weaning period. Taking the average temperature for a 21-day period (Tavg21) as the heat load of choice, the expected weight at 23 °C, i.e., under a heat load equivalent to the moderate thermotolerance threshold +2 °C, would be 250 g less, and this loss would reach 1.85 kg at 29 °C, equivalent to a heat load of +2 °C above the severe thermotolerance threshold. This represents a substantial percentage of weight of kids in this breed, indicating that this loss in meat production associated with HS is not marginal. Nevertheless, some refinements would be needed in order to obtain an accurate estimate of the overall loss in meat production associated with HS. For example, the model assumed that the loss in weight associated with HS is the same regardless of the age of the animal, which might not be realistic, therefore, more data would be needed to accurately solve more complex models. In addition, kids during the pre-weaning period are in the growing phase and are thus likely to have compensatory growth after the HS ceases.

Overall, our results show that exposing growing kids to ambient temperatures out of the thermoneutral zone will affect their performance and significantly hamper their production. Despite the previously discussed adaptive mechanisms of local breeds, prolonged exposure to heat stress can surpass an animal’s capacity to maintain homeostasis, leading to substantial productivity declines. It should be noted that, in our study, the kids under heat stress were born in the dry season. Our findings agree with the results of Phillips [6], who reported that goats born during the rainy season had a greater birth weight and reached the weaning stage at a faster rate in contrast to goats born during the dry season. However, this weight loss could also be attributed to the direct effects of high temperatures on the individual, with a reduction in feed intake, which directly translates to lower energy and nutrient intake, to a negative energy balance, and, consequently, to weight loss [7]. Moreover, heat stress increases the energy demands for physiological processes, which further depletes the energy reserves [8]. In agreement with our findings, Hamzaoui et al. [9] showed that heat-stressed goats exhibited a significant reduction in body weight compared to those kept in thermoneutral conditions. Similarly, a study by Salama et al. [10] reported a decrease in the average daily gain and body weight in heat-stressed goats, highlighting the adverse effects of high ambient temperatures on growth performance.

Taking into account individual deviations from the average pattern of response of weight to increasing environmental heat, an enlarged variability of weights was observed under HS conditions compared with thermoneutrality. In our case, the estimated variability of weights became larger as HS severity increased. A reduction in the variability of productive traits under unfavorable environments might have been expected, because a restrictive environment could hamper the full expression of the genetic potential for the production of animals. On the other hand, higher variability under restrictive conditions could be the result of the animals showing existing variability in adaptation to the harsh environments, which would add up to the basal variability of the potential for production under favorable conditions. The results from the literature relative to the effect of HS on productive traits are in fact heterogeneous. For beef cattle, the variability of weaning weight increased with heat load in the study of Bradford et al. [11]; however, a decreasing and then stable estimated variance of weight as temperature increased was found by Santana et al. [12]. The same heterogeneity of results has been reported for the variability of milk production under increasing heat. A reduced variability under HS for milk productive traits has been observed by Brügemann et al. [13] and Hammami et al. [14], but higher variability under high heat loads was reported by Ravagnolo and Misztal [15] and Bernabucci et al. [16]. In our case, the fact that high growth (according to the individual estimated values for intercepts) was positively correlated (results in Table 4) with heat tolerance (from the estimated slopes of change in weight under HS) points at animals with a higher weight potential being better adapted to the productive conditions. Thus, both weight and slopes of change in weight under HS conditions could be used as indicators of the adaptation of animals to high temperatures. However, the fact that correlations between estimated individual values for weight level and for slopes decreased as HS severity increased ought to be taken into account for a selection program. A similar positive association was found by Bradford et al. [11] for the genetic components of weight and slopes, but a negative genetic correlation between weight potential and the slope of change under HS was reported by Santana et al. [12]. Slowing growth might be an adaptive response to heat in animals with high growth rates, which might imply a greater production of internal heat. This phenomenon has been discussed by Berman [17] in dairy cattle. Nevertheless, growth is considered to be a less intense system in terms of energy demand and heat release than milk production from highly selected breeds, and antagonistic relationships between growth potential and heat tolerance would not be expected in a local unselected breed such as the Tunisian goat.

In addition to the substantial variability in weights, variability in individual slopes was also found in this study. The amplitude of the range of slopes for individual animals was smaller for Tmin compared with Tavg or Tmax for all periods, pointing again to Tmin as a less suitable heat load definition in terms of allowing a full display of the reaction to HS. The percentage of the estimated variance of slopes, with respect to the estimated variance of levels under HS, was around 1%/°C (results not shown). This is smaller than the ratio found by Santana et al. [12], ranging from 5 to 25%/°C for different beef cattle breeds. The relative lower variability in slopes, with respect to the variability in weights, would point to a less efficient selection for that trait.

## 5. Conclusions

In this study, we evaluated the effect of heat stress on the growth of local goat kids from birth to weaning, focusing on body weight as a critical economic trait in goat production. Our findings underscore the significant negative impact of heat stress on goat performance, evidenced by reductions in body weight under the conditions of moderate and acute heat stress. The acute stress led to relevant weight losses of up to 0.5 kg/°C per animal. The models in which cubic Legendre polynomials were fitted using average daily temperature as a measure of heat load are preferred to display the impact of heat stress on body weight. The average daily temperatures proved to be more effective predictors of heat stress impacts compared to the maximum or minimum temperatures alone, highlighting their utility in modeling weight responses in goats. The variability observed in weight and weight change under HS provides a favorable background for the genetic selection of heat tolerance. The animals with larger weights also showed more stable weights under HS. This fact, together with the higher variability in weights found under HS, make the weight records obtained under HS valuable indicators of heat tolerance in this population. These insights are crucial for informing selective breeding strategies aimed at enhancing livestock resilience to environmental stressors like heat, particularly in arid regions where such conditions are prevalent.

## Figures and Tables

**Table 1:** Minimum (Min), percentiles 10 and 90 (P10, P90), mean, standard deviation (SD) and maximum (Max) for the number of weights available per kid (Nw/k), age at weighing, weight, averages of minimum (Tmin), maximum (Tmax), and average (Tavg) daily temperatures on the day of weighing (0), and averages of those values for the 7, 14, and 21 previous days.

Variables	Min	P10	Mean	SD	P90	Max
Nw/k	2	3	7.1	4.0	11	23
Age	1	15	72.3	47.6	143	200
Weight	1.4	3.2	7.	3.9	13.3	23.4
Tmin0	0.0	3.0	8.4	5.1	16.0	29.0
Tmin7	0.3	3.8	8.9	4.9	15.8	35.3
Tmin14	2.1	4.5	8.8	4.6	15.8	31.4
Tmin21	2.1	4.5	8.7	4.3	15.8	31.2
Tmax0	10.5	16.0	23.0	6.2	33.9	36.0
Tmax7	13.0	15.9	21.9	5.6	30.0	40.0
Tmax14	13.0	16.0	21.7	5.2	29.2	36.7
Tmax21	13.6	16.0	21.5	5.0	29.5	34.0
Tavg0	5.8	10.0	15.8	5.2	23.5	29.3
Tavg7	6.7	10.0	15.2	5.0	22.5	35.7
Tavg14	8.2	10.4	15.1	4.7	22.6	31.7
Tavg21	9.0	10.2	14.9	4.5	22.6	31.5

**Table 2:** Heat stress thresholds (HST in °C) obtained from change points in the trajectory of the response of weight to temperature and slopes of decay in weight per degree of temperature (Slp in kg/°C) beyond HST together with associated weight loss under moderate (HS1 = HST1 + 2 °C) and severe (HS2 + 2 °C) heat stress for averages of minimum (Tmin), maximum (Tmax), and average (Tavg) daily temperatures during 7, 14, and 21 days prior to the date of weighing for a cubic polynomial response function.

		HS Thresholds and Slopes of Decay				Expected Weight Loss	
Temperature	Period	HST1	Slp1	HST2	Slp2	HS1	HS2
Tmin	7	13.6	−0.075	25.4	−0.228	−0.15	−1.34
	14	8.6	−0.092	17.2	−0.235	−0.18	−1.26
	21	7.6	−0.090	15.3	−0.259	−0.18	−1.21
Tmax	7	30.6	−0.105	36.3	−0.291	−0.21	−1.18
	14	27.4	−0.080	32.4	−0.265	−0.16	−0.93
	21	25.6	−0.075	30.5	−0.330	−0.15	−1.03
Tavg	7	23.7	−0.135	30.5	−0.430	−0.27	−1.78
	14	18.6	−0.074	25.5	−0.350	−0.15	−1.21
	21	17.7	−0.125	25.3	−0.450	−0.25	−1.85

**Table 3:** Summary statistics (minimum (Min), percentiles 10 (P10) and 90 (P90), median, and maximum (Max)) for the estimated breeding values of individual slopes of change in weight (kg/°C) at moderate (HST1 + 2 °C) and acute (HST2 + 2 °C) values of the temperature under models fitting quadratic (quad) or cubic Legendre polynomials on the average of minimum (Tmin), maximum (Tmax), and average (Tavg) daily temperatures during 7, 14, and 21 days prior to the date of weighing.

Heat Load	HS Level	Temp.	Min	P10	Median	P90	Max
Tmin7	HS1	16	−0.41	−0.18	0.00	0.19	0.54
	HS2	27	−1.31	−0.47	0.00	0.48	1.39
Tmin14	HS1	11	−0.40	−0.15	0.00	0.15	0.55
	HS2	19	−0.91	−0.37	−0.01	0.38	1.01
Tmin21	HS1	10	−0.38	−0.12	0.00	0.12	0.41
	HS2	17	−0.79	−0.33	−0.02	0.35	0.95
Tmax7	HS1	33	−1.10	−0.43	−0.02	0.48	1.19
	HS2	38	−2.30	−0.64	0.01	0.72	1.72
Tmax14	HS1	29	−0.97	−0.38	−0.03	0.42	1.00
	HS2	34	−1.88	−0.55	−0.03	0.60	1.36
Tmax21	HS1	28	−1.13	−0.46	−0.03	0.50	1.24
	HS2	32	−1.52	−0.54	0.00	0.58	1.34
Tavg7	HS1	26	−1.03	−0.41	−0.01	0.45	1.03
	HS2	33	−2.48	−0.66	0.01	0.69	2.16
Tavg14	HS1	21	−0.82	−0.38	−0.02	0.40	1.03
	HS2	28	−1.64	−0.53	−0.01	0.52	1.37
Tavg21	HS1	20	−0.77	−0.35	−0.02	0.41	0.97
	HS2	27	−1.28	−0.49	−0.01	0.50	1.16

**Table 4:** Overall rank correlations between predicted values for intercepts (Int) and slopes of change in weight per degree of increase in heat load under mild (SlpHS1) and severe (SlpHS2) conditions for alternative definitions of heat load (Tmin, Tmax, and Tavg) and periods of days (7, 14, and 21) prior to the date of weight recording.

Temperature	Period	Int-SlpHS1	Int-SlpHS2	SlpHS1-SlpHS2
Tmin	7	0.92	0.70	0.79
	14	0.84	0.70	0.56
	21	0.82	0.80	0.70
Tmax	7	0.74	0.53	0.95
	14	0.74	0.40	0.89
	21	0.78	0.32	0.79
Tavg	7	0.78	0.50	0.91
	14	0.88	0.35	0.64
	21	0.89	0.12	0.31

## Data Availability

All data generated or analyzed during this study are included in the published article. For further details, please contact the corresponding author.

## References

[1] Ouni M., Najari S., Gaddour A., Andrea C. (2007). Early growth of morphometric traits of local goat population in Tunisian arid zone. J. Bio. Sci..

[2] Atoui A., Carabano M.J.C., Lasoued M., Laroussi M., Abdennebi M., Tlahig S., Ben Salem F., Najari S. (2023). Prediction of birth weight using body measurements of local caprine population kids raised in a low-input breeding mode under arid environment. Trop. Anim. Health Prod..

[3] Najari S. (2005). Caractérisation Zootechnique et Génétique d’une Population Caprine. Cas de La Population Caprine Locale des Régions Arides Tunisiennes. Ph.D. Thesis.

[4] Atoui A., Carabaño M.J., Abdenebi M., Najari S. (2022). On the modelling of weights of kids to enhance growth in a local goat population under Tunisian arid conditions. The maternal effects. Trop. Anim. Health Prod..

[5] Muggeo V.M.R. (2008). Segmented: An R package to fit regression models with broken-line relationships. R News.

[6] Phillips C.J.C. (2004). Heat stress in goats. Small Rumin. Res..

[7] Silanikove N. (2000). Effects of heat stress on the welfare of extensively managed domestic ruminants. Livest. Prod. Sci..

[8] Marai I.F.M., El-Darawany A.A., Fadiel A., Abdel-Hafez M.A.M. (2007). Physiological traits as affected by heat stress in sheep. A review. Small Rumin. Res..

[9] Hamzaoui S., Salama A.A.K., Caja G. (2013). Effect of heat stress on the dairy cattle: Responses, mechanisms, and management: A review. Trop. Anim. Health Prod..

[10] Salama A.A.K., Caja G., Hamzaoui S., Badaoui B., Castro-Costa A., Façanha D.A.E., Guilhermino M.M., Bozzi R. (2014). Different levels of response to heat stress in dairy goats. Small Rumin. Res..

[11] Bradford H.L., Fragomeni B.O., Bertrand J.K., Lourenco D.A.L., Misztal L. (2016). Regional and seasonal analyses of weights in growing Angus cattle. J. Anim. Sci..

[12] Santana M.L., Bignardi A.B., Eler J.P., Ferraz J.B.S. (2015). Genetic variation of the weaning weight of beef cattle as a function of accumulated heat stress. J. Anim. Breed. Genet..

[13] Brügemann K., Gernand E., Borstel U.U., Konig S. (2011). Genetic analyses of protein yield in dairy cows applying random regression models withtime-dependent and temperature x humidity-dependent covariates. J. Dairy Sci..

[14] Hammami H., Vandenplas J., Vanrobays M.L., Rekik B., Bastin C., Gengler N. (2015). Genetic analysis of heat stress effects on yield traits, udder health, and fatty acids of Walloon Holstein cows. J. Dairy Sci..

[15] Ravagnolo O., Misztal I. (2000). Genetic component of heat stress in dairy cattle, parameter estimation. J. Dairy Sci..

[16] Bernabucci U., Biffani S., Buggiotti L., Vitali A., Lacetera N., Nardone A. (2014). The effects of heat stress in Italian Holstein dairy cattle. J. Dairy Sci..

[17] Berman A. (2011). Thermal effects on fertility of dairy cattle. Animal.

